# Improving the state-of-the-art in Thai semantic similarity using distributional semantics and ontological information

**DOI:** 10.1371/journal.pone.0246751

**Published:** 2021-02-17

**Authors:** Ponrudee Netisopakul, Gerhard Wohlgenannt, Aleksei Pulich, Zar Zar Hlaing

**Affiliations:** 1 Faculty of Information Technology, King Mongkut’s Institute of Technology Ladkrabang (KMITL), Bangkok, Thailand; 2 Faculty of Software Engineering and Computer Systems, ITMO University, St. Petersburg, Russia; University of Birmingham, UNITED KINGDOM

## Abstract

Research into semantic similarity has a long history in lexical semantics, and it has applications in many natural language processing (NLP) tasks like word sense disambiguation or machine translation. The task of calculating semantic similarity is usually presented in the form of datasets which contain word pairs and a human-assigned similarity score. Algorithms are then evaluated by their ability to approximate the gold standard similarity scores. Many such datasets, with different characteristics, have been created for English language. Recently, four of those were transformed to Thai language versions, namely WordSim-353, SimLex-999, SemEval-2017-500, and R&G-65. Given those four datasets, in this work we aim to improve the previous baseline evaluations for Thai semantic similarity and solve challenges of unsegmented Asian languages (particularly the high fraction of out-of-vocabulary (OOV) dataset terms). To this end we apply and integrate different strategies to compute similarity, including traditional word-level embeddings, subword-unit embeddings, and ontological or hybrid sources like WordNet and ConceptNet. With our best model, which combines self-trained fastText subword embeddings with ConceptNet Numberbatch, we managed to raise the state-of-the-art, measured with the harmonic mean of Pearson on Spearman *ρ*, by a large margin from 0.356 to 0.688 for TH-WordSim-353, from 0.286 to 0.769 for TH-SemEval-500, from 0.397 to 0.717 for TH-SimLex-999, and from 0.505 to 0.901 for TWS-65.

## Introduction

The ability to understand semantic similarity between given terms is strongly related with understanding natural language in general [1]. Therefore, semantic similarity is a very popular research area in lexical semantics [2]. For evaluating the capability of a method or model on the task, typically manually curated semantic similarity datasets are used. The datasets are general-domain and allow the study of global word usage. Generally, those datasets contain word (or n-gram) pairs, and a similarity score for each pair assigned by human experts. The datasets go back to RG-65 [3], created in 1965, including only 65 word pairs. Newer datasets are much larger in size, and differ with regards to the definition of similarity (relatedness vs. similarity [4], see Section Related Work), the inclusion of n-grams and named entities [2], and other aspects. Word similarity has applications in many NLP areas, such as word sense disambiguation [5], machine translation [6], or question answering [7]. Moreover, there are evaluation campaigns like SemEval 2017 (Task 2) solely dedicated to improving the state-of-the-art on the semantic similarity task.

Word representations have gained a lot of interest in the last years due to new advancements regarding the use of neural networks to learn low-dimensional, dense vector representation models known as word embeddings, for example with the word2vec [8] toolkit. Word embeddings are also commonly used as input in natural language processing (NLP) tasks when using machine learning, esp. deep learning architectures. A good embedding model provides vector representations for words where the (geometric) relation between two vectors reflects the linguistic relation between the two words [9], it aims to capture semantic and syntactic similarities between words [10]. In the evaluation of word embeddings, there is generally a distinction between intrinsic and extrinsic evaluation methods. While in intrinsic evaluation vectors from word embeddings are directly compared with human judgement on word relations, extrinsic evaluation measures the impact of word vector features in supervised machine learning used in downstream NLP tasks [11]. To evaluate the quality of an embedding model, semantic word similarity is generally accepted as the most direct intrinsic evaluation measure for word representations [2, 9]. During word embedding model training, the word similarity task can be applied to estimate the embedding model quality and for hyperparameter tuning [10, 12].

Although the word semantic similarity task is very popular for evaluating word embeddings, as it is fast and computationally inexpensive, practitioners need to be aware of potential pitfalls, for example that high scores on intrinsic evaluation do not guarantee best results in the downstream application [13]. However, downstream (extrinsic) evaluation is often expensive or impractical (due to missing evaluation datasets), so that intrinsic evaluation at least provides helpful evidence and direction for comparing models and algorithms. Bakarov [11] provided an in-depth survey of existing strategies for the evaluation of word embeddings.

Regarding Thai word embeddings, there are only few pretrained Thai word embedding models available online. Those are fastText [14], Thai2vec [15], ft-wiki [16], and Kyu-ft and Kyu-w2v [17]. Previous work evaluated these models against four human-rated Thai similarity datasets [18]. Those Thai datasets are: TH-WordSim-353, TH-SemEval-500, TH-SimLex-999 and TWS-65 [19], all of which are based on English datasets, which were translated, and then the similarity scores were re-assigned in the target language.

The authors of the previous evaluations reported a number of difficulties with the pretrained models, first of all, a high number of out-of-vocabulary (OOV) terms [18]. The problem is related to the peculiarities of Thai language, which were discussed at length in Netisopakul and Wohlgenannt [20]. In brief, firstly, written Thai language, like some other Asian languages (e.g. Lao, Burmese, Cambodian), is a continuous conjugated text without spaces between words. Secondly, there is no common agreement on what constitutes a *basic term*, even among Thai NLP experts. Thirdly, most Thai terms are composed of multiple *basic terms*, for example, “river” in Thai literally is composed of the two terms “mother+water”, “student” is “person+learn” and so on. This third aspect has the largest effect on the Thai word similarity datasets, and the OOV problem. Often a basic word in English, when translated, becomes a compound term in Thai. That is, the translated Thai term can be decomposed into two or more *basic terms* in Thai—depending also on the word segmentation tool applied. However, the meaning of decomposed terms are not the same as the compound term, although they may semantically contribute to some aspects of the compound term. All these factors make the Thai word segmentation task a crucial step but not at all an easy one. The results of the subsequent Thai NLP tasks are greatly affected by word segmentation. Examples of word segmentation from the dataset are words such as ‘blizzard’ and ‘avalanche’, which translated to Thai OOV words namely ‘Phayuhima’ (’Phayu = Storm’+’Hima = Snow’) and ‘Himathlm’ (’Hima = Snow’+’Thlm = Collapse’), respectively. So, the word segmentation tool will have to segment ‘Phayuhima’ into ‘Phayu’ and ‘hima’, and segment ‘Himathlm’ into ‘Hima’ and ‘thlm’. Consequently, the reduction of the number of OOV terms is one of the main issues to improve the evaluation metrics.

The goal of this research is to improve the state-of-the-art in semantic similarity for the Thai language. As evaluation score for semantic similarity, most authors (see Section Related Work) used Pearson’s or Spearman’s *ρ*, or the harmonic mean of the two. We report all three scores. To achieve our goal, we employ methods to solve the OOV problem, and inspired by the best performing systems in the SemEval-2017 (Task 2) competition, we also combine word embedding models with information from structured data sources, namely WordNet. Moreover, we use ConceptNet Numberbatch [21], which is built from an ensemble of traditional word embeddings and the ConceptNet knowledge graph using retrofitting [22].

**Fig 1:**
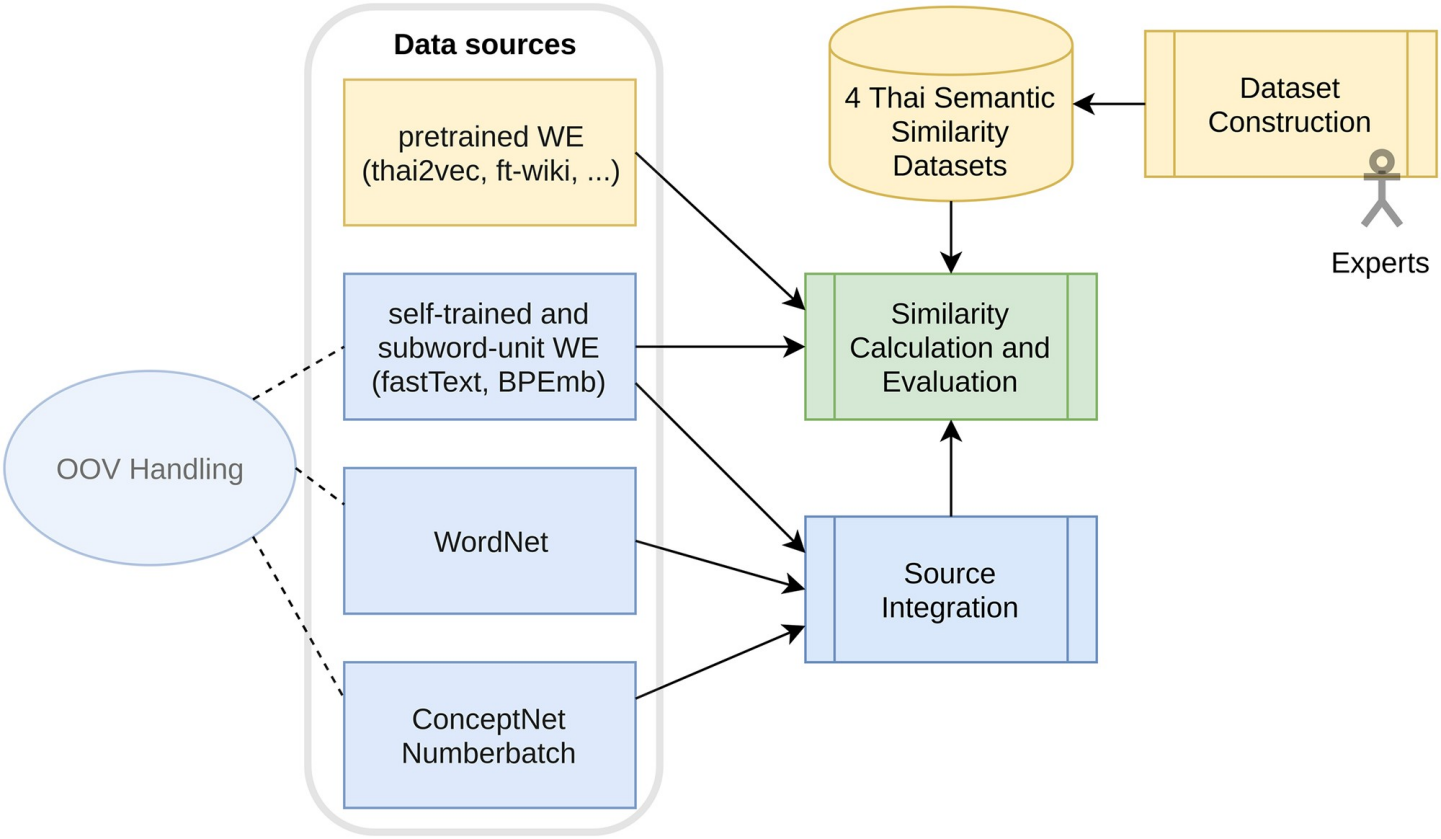
System overview. Main steps in Thai semantic similarity conducted in previous work (yellow background), and this work (blue background).

For easier orientation, Fig 1 provides on overview of steps taken in previous work (graph shapes with yellow background color), and in this paper (blue background color). “Similarity Calculation and Evaluation” is a crucial part in current and previous work.

Summarizing the main results of this work, we apply different strategies to improve over the existing state-of-the-art [18]. Firstly, training our own models with word2vec [8] and fastText [23] improves the metrics slightly, but does not solve the OOV problem. In a second iteration, we apply the Thai deepcut word tokenizer [24] both on the corpus and dataset strings, which eliminates a large portion of OOV words and improves the evaluation metrics. Thirdly, the use of subword-unit embeddings in the form of BPEmb [25] and fastText with subword-units [23] effectively solves the OOV problem. Next, we experiment with the similarity functions available in Thai WordNet [26]. Thai WordNet by itself cannot compete with the embedding models regarding the evaluation metrics, but a combined (ensemble) approach improves the best overall results. And finally, using ConceptNet Numberbatch in combination with fastText helps to generate the clearly best results overall.

In this work, we raise the average evaluation score over all datasets from 0.38 (previous work) to 0.77. Human-level agreement on the datasets is in the range of 0.73 to 0.83, so the work provided is a large step towards human-level performance on the Thai semantic similarity task. In conclusion, this work is the first in-depth and large-scale study of semantic similarity between terms for Thai language, and discusses and evaluates solutions to the important problem of OOV words in Thai.

The remainder of the paper is organized as follows: In Section Related Work we present related work in the field, followed by a description of the datasets, models, and integration strategies used in this work (Section Datasets and models). In Section Evaluation we describe the experiments and results for the different strategies, including the evaluation setup. Finally, Section Conclusion concludes the work.

## Related work

For English language, a number of standard word similarity datasets are available. WordSim-353 [27] and MEN [28] are two popular datasets that do not distinguish between relatedness and similarity in their similarity assignment. SimLex-999 [4] on the other hand aims to measure similarity more strictly, in contrast to relatedness. While a dataset like WordSim-353 would give a word pair such as *weather-forecast* a high similarity score, the score would be low in SimLex-999. The very recent dataset of the SemEval 2017 (task 2) competition (SemEval-500) [2] introduced multi-word expressions and named entities into the dataset. With the exception of SemEval-500, which was released in 5 languages, most datasets were originally created only in English language versions. In the last decade there has been considerable work to translate datasets into other languages.

For example, Akhtar et al. [29] translated the RG-65 and WordSim-353 datasets into six Indian languages, Panchenko et al. [30] translated RG-65, MC-30 and WordSim-353 to Russian, and Chen and Ma [31] translated SimLex-999 to Chinese.

Many modern NLP systems represent words in the form of dense floating-point vectors, with a small and fixed dimensionality (for example 300 dimensions). The vectors for the words in the vocabulary are trained so that semantically similar words will have similar vectors. Then, a similarity score between two terms can be computed simply eg. with the cosine of the angle in vector space. There are generally two ways to create word representations in vector form, count-based, and prediction-based, methods. Count-based models start from co-occurrence counts (for example a term-document or term-term matrix). Typically counts are re-weighted, or dimensionality-reduction techniques like SVD or PCA are applied to the raw co-occurrence counts in order to raise performance [32]. A new generation of distributional semantics models (prediction-based models) frame vector generation as a supervised task where the weights of the word vectors are set to predict the probability of a word appearing in a specific context. Based on the distributional hypothesis [33], which states that similar words appear in similar contexts, the learning algorithm is supposed to assign similar vectors to similar words. Well-known examples for prediction-based word vector construction are word2vec [8] and fastText [23].

Most traditional distributional semantics models operate on a word level. Depending on the vocabulary size, this leads to OOV words, and furthermore, vector representations of rare words tend to be of low quality. Moreover, in languages with a rich morphology it is far from clear what actually counts as a word [34]. In the last years a number of embedding algorithms appeared which model language on a character or subword-unit level, for example fastText [23] or BPE [35]. Such models can share subword unit information across words, and therefore better represent morphological variants and rare words [36].

In contrast to the *global* word embeddings used here, depending on the task, many NLP applications also use contextualized encodings of text fragments typically either based on recurrent neural network models, for example [37], or lately mostly based on the transformer architecture [38, 39]. Such models provide state-of-the-art performance on tasks such as paraphrasing or text classification, but are not designed for the traditional term similarity task, which we study in this paper.

## Datasets and models

As stated in the introduction section, the task of semantic similarity between words has a long tradition in lexical semantics, and is the most widely used method of intrinsic evaluation of word embedding models. Our experiments are focused on improving the state-of-the-art on the task for Thai language, therefore we use the datasets available in Thai. In this section, we first give an overview of the datasets (Section Datasets), and in Section Models and algorithms we introduce the techniques which are later applied on the semantic similarity task in the evaluation section. Those techniques include different types of word embedding algorithms, as well as a structured data source (WordNet) and a hybrid (ConceptNet). Finally, we introduce the evaluation metrics, and a user-friendly tool to evaluate the models with regards to the datasets.

### Datasets

Four datasets for semantic similarity exist in Thai: TWS65 [19], TH-WordSim-353, TH-SemEval-500, and TH-SimLex-999 [18]. All datasets were created by translating and re-rating the English-language originals. Based on best practice in similar translation efforts, Netisopakul et al. [18] employed two translators for the word pairs of the datasets, and in case of disagreement between the translators, a third one decided. After translation, the terms were re-rated in the target language—as translation affects the meaning of terms. For the small TWS65 dataset, Osathanunkul et al. [19] used 40 raters per term pair, for the other datasets 10 to 16 raters suggested similarity scores for each term pair. The final datasets uses the average human ratings as gold standard similarity score for the word pairs.

**Fig 2:**
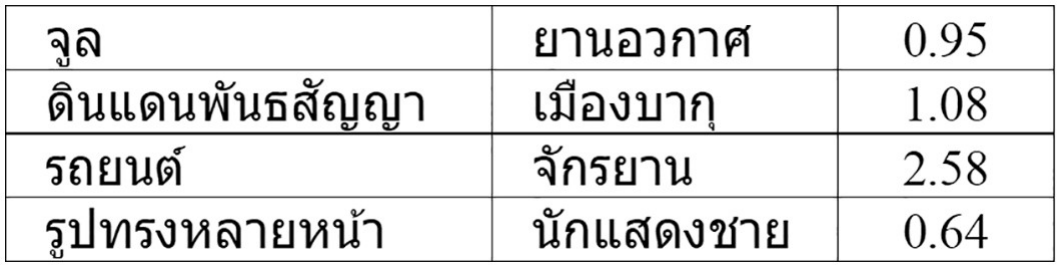
First four lines of TH-SemEval-500.

The datasets are available online [40] as .csv files, and include the two terms and the similarity score. To give an example, Fig 2 presents the first four entries in the Thai SemEval-500 dataset. The third pair in the figure is *“car,bicycle”* and has a much higher similarity score than for example the first pair (*Joule,spacecraft*).

The inter-annotator agreement (IAA) between humans with regards to the similarity scores is a human-level baseline for the algorithms. Table 1 presents an overview of the four datasets, including the number of term pairs, the IAA, and the rating scale/interval. For all rating scales, higher numbers indicate higher similarity.

**Table 1:** Overview of Thai semantic similarity datasets, including number of word pairs, human inter-annotator agreement, and rating interval.

	TH-WordSim-353	TH-SemEval-500	TH-SimLex-999	TWS-65
Number of word pairs	353	500	999	65
Inter-annotator agreement	0.732	0.800	0.826	Not reported
Rating scale	0−10	0−4	0−10	0−4

#### TH-WordSim-353

This dataset is based on the very popular WordSim-353 [27], a dataset which measures primarily the relatedness between terms rather than similarity. To exemplify the distinction, *coffee* and *cup* are strongly related, but have low similarity in their characteristics.

#### TH-SemEval-500

The original version of this dataset was published in 2017 [2], and is designed to be very challenging by including word pairs from 34 domains such as chemistry, computing and culture. Furthermore, the dataset contains multi-word terms and named entities from any of the 34 domains. Also, there is a distinction between similarity and relatedness, in dataset construction the raters were instructed to rate similarity higher than relatedness.

#### TH-SimLex-999

In contrast to WordSim-353, this dataset [4] is designed to capture similarity between terms and not just relatedness. The dataset is challenging, and it includes a high number of antonym pairs. The 999 word pairs result from 666 noun, 222 verb and 111 adjective pairs. All terms in the original dataset are taken from the English version of WordNet [41],

#### TWS-65

Finally, TWS-65 is based on the classical dataset from year 1965 created by Rubenstein and Goodenough [3]. The dataset is very small (65 word pairs), and focuses primarily on similarity, not on relatedness.

### Models and algorithms

In this work, we experiment with different approaches and models to improve the state-of-the-art in Thai semantic similarity. Firstly, in previous work, one of the difficulties found with Thai language when using pretrained word embeddings was the high number of out-of-vocabulary (OOV) words. We tackle this problem by training our own models with word2vec and fastText, and by using subword-unit embeddings like BPE. On the other hand, in competitions in the field (like SemEval-2017, Task 2 [2]) the best performing systems often combine word embeddings with structured knowledge sources—we employ Thai WordNet and ConceptNet as structured (or hybrid) sources.

#### word2vec

The word2vec model [8] is based on a shallow architecture with only two network layers, which allows to efficiently train on very large text corpora. The training goal is to reconstruct the linguistic context of words. Word2vec contains two algorithms, continuous-bag-of-words (CBOW) and skip-gram. In CBOW, the model predicts the surrounding words from a given word, while in skip-gram mode the surrounding word context is used to predict the target word. One of the most important hyperparameters is the *word window size*, which defines the context to be used in prediction, for example, two words to the left and two to the right. Further, the *dimension* parameter specifies the size of the resulting word vectors.

#### fastText

fastText [23] is an extension of the word2vec model. In contrast to word2vec, it treats words as being composed of character n-grams instead of atomic entities. The tool can either save the word vectors to files (such as word2vec), or it can generate models that include subword-unit information. The subword-unit information facilitates prediction of OOV words by composing the word vector from its subword-unit parts, and thereby helps to solve the OOV problem. We make use of this feature in the evaluation section. fastText is also known for its large range of pre-trained models in 294 languages.

#### BPEmb

BPE [35] is another recent approach to generate subword embeddings, and to solve the OOV issue. Similar to fastText, the approach uses byte-pair encodings to leverage subword information without the need for tokenization or morphological analysis. BPEmb [25] provides pre-trained BPE subword embeddings in 275 languages trained on Wikipedia [42]. The pretrained embeddings are available in many vocabulary sizes, from 1K to 200K. Depending on the vocabulary size used, a word like *Melfordshire* might be decomposed into the subwords *Mel ford shire*. Generally, with a small vocabulary size, words are often split into many subwords, while with a larger vocabulary, frequent words will not be split. Byte-pair encoding leads to a dramatically reduced model size, depending on the chosen vocabulary size and vector dimensions.

#### Thai WordNet

WordNet [41] is a very popular lexical database for English. Nouns, verbs, adjectives and adverbs are grouped into so-called *synsets*, which are (near) synonyms expressing a particular concept. The synsets are interlinked with different semantic relation types such as hypernymy, meronymy or antonymy into a large network structure (including around 117K synsets). Thai WordNet [26] was created in a semi-automatic way based on the English Princeton WordNet using a bi-lingual dictionary and manual translation checking. In the experiments, we use the Thai WordNet version included in the PyThaiNLP [43] toolkit for Thai language. The central feature of WordNet relevant to this work are various *similarity* functions between terms. Thai WordNet includes the following functions: *path_similarity, lch_similarity, wup_similarity*. The *path_similarity* metric is based on the shortest path between two synsets within the is-a (hypernymy) taxonomy. *wup_similarity* (Wu-Palmer similarity) denotes the similarity of two terms depending on their depth in the taxonomy and the depth of the least common subsumer node. Finally, *lch_similarity* is only supported for synsets with the same POS-tag, which we cannot guarantee for the dataset word pairs.

#### ConceptNet

ConceptNet [44] is a knowledge graph in the Linked Open Data (LOD) format, and connects words and phrases of natural language with labeled edges [21]. The knowledge in ConceptNet stems from a multitude of sources such as crowd-sourcing, games with a purpose and experts. The goal of ConceptNet is to provide general knowledge needed for language understanding which can be applied for example in NLP applications.

*ConceptNet Numberbatch* [45] is a set of word embeddings that combine ConceptNet with distributional sources such as word2vec [8] and GloVe [46] using a variation of retrofitting [22]. The embeddings therefore are informed both by the pure contextual knowledge of distributional models and by the structured common sense knowledge of ConceptNet. Moreover, Numberbatch has a multilingual design with many different languages sharing one common semantic space. The number of Thai terms (marked with /c/th/), is around 95K in the current version (19.08.) of Numberbatch. ConceptNet took first place in two *Semantic Word Similarity* tasks at SemEval 2017 [2]. Finally, ConceptNet provides its own OOV strategy which is as follows: If a term is not found in the vocabulary, remove the last letter at end, and take the average vector of all words in the model vocabulary starting with the truncated term.

ConceptNet is accessible via a public JSON-LD API [47], and provides an API method to compute the *relatedness* of two terms. Alternatively, the Numberbatch embeddings can be downloaded from GitHub and used locally—which is the strategy we applied.

### Implementation

We implement a tool which allows to easily evaluate a Thai word embedding model with respect to the datasets. The tool is forked from an existing tool designed to evaluate English language datasets and models [48]. Our adapted and extended version is available on GitHub [49]. To evaluate a word embedding model with regards to the four datasets, it is sufficient to simply provide the model file path to the evaluation script.

In tool adaption, first we integrate the Thai semantic similarity datasets into the tool. In previous work we discuss a simple approach to the problem of OOV words by splitting the dataset terms into parts (with the deepcut tokenizer) and using the sum of the vectors of those parts as the word vector. For implementation details see Netisopakul et al. [18].

The main update to the tool in this work concerns the experiments with structured and hybrid sources (WordNet and ConceptNet). In addition to computing a similarity score for a word pair based on a word embedding model, the tool can compute WordNet- and ConceptNet-based similarity. We tested the WordNet *path_similarity* and *wup_similarity* similarity measures (see above), and decided to rely on *path_similarity* only, as it consistently provided better results. Furthermore, for computing the *path_similarity* it is necessary to select a distinct WordNet synset from the number of synsets where the word is present. Here we tried two variants: (i) in the “simple variant” we choose the first synset (if any) for both terms of the input word pair; (ii) the “most similiar” variant computes the *path_similarity* between all possible combinations of synsets of the two input terms, and then selects the highest similarity score.

For ConceptNet, we first downloaded the vector models from GitHub [45] and then implemented the ConceptNet OOV strategy of word truncation (see above) into our tool.

For the integration of the scores provided by the word embeddings and WordNet (or ConceptNet), we apply two slightly different approaches. In both cases, we use a coefficient *α* to determine which portion of the word embedding (WE) score and which portion of the WordNet or ConceptNet (WN) similarity ends up in the final score. This basic idea is in alignment with approaches used by some SemEval 2017 (task 2) contenders [50]. So the final score in Eq 1 for a word pair *i* of the dataset is simply a weighted combination of the two parts, and in the evaluations we test different values to find a good *α* coefficient.

$$\begin{array}{c} \text{Final-Score}=\alpha *WE_{i}+\left(1-\alpha \right)*WN_{i} \end{array}$$ (1)

Using this basic formula, we have to consider a few observations: For 11%-37% (depending on the dataset, see evaluation section) of word pairs, no WordNet path could be found (mainly because the terms were OOV in WordNet). Secondly, the word embedding and WordNet scores have different distributions and scales of their similarity scores. Given this situation, we evaluate two methods of transforming the scores in order to be able to apply Eq 1: using the average WordNet score in cases of OOV terms (*Method 1*), and normalizing the distributions (*Method 2*).

Eq 2 shows how we compute the WordNet or ConceptNet (WN) score for a word pair with *Method 1*. First, in the case of WordNet, we compute the average *path_similarity* per dataset for all word pairs *j* for which WordNet paths are found. If for the given word pair *i* no WordNet path is found, then we use the average score. Otherwise, the actual WordNet score is used. The final score is then determined with Eq 1.

$$\begin{array}{c} {\text{M1-WN}}_{i}=\{\begin{array}{cc} \frac{1}{n}\sum_{j=1}^{n}WN_{j}, & \text{if}\;\text{no}\;\text{WordNet}\;\text{path}\;\text{found}.\\ WN_{i}, & \text{otherwise} \end{array} \end{array}$$ (2)

For *Method 2* we approach the problem of missing WordNet paths in a slightly different way. We normalize (per dataset) both the list of WE-scores and WN-scores to have a mean of 0 and a standard deviation of 1. If we do not find a WordNet *path_similarity* of a word pair, we use only the word embedding (WE) score, which equals to setting *α* = 1 in this situation. For the other pairs, we simply input the (normalized) scores into Eq 1. With regards to ConceptNet we use the same strategies for integration (Method 1 and 2).

## Evaluation

As mentioned in Section Introduction, as *evaluation metric* we use Pearson’s *ρ*, Spearman’s *ρ*, and the harmonic mean of the two—in conformance with Camacho-Collados et al. [2]. Netisopakul et al. [18] evaluated existing pre-trained word embedding models on the word similarity tasks for the four datasets. The best results when using the datasets “as is”, were between 0.29 (for TH-SemEval-500) and 0.50 (for TWS65). The authors also experimented with applying deepcut tokenization to the dataset terms in order to reduce the fraction of out-of-vocabulary (OOV), which helped to raise the results in the range of 0.39 (TH-SemEval-500) to 0.56 (TWS65). Those results from previous work are used as baseline in the evaluations presented here. As the Thai evaluation datasets are very recent at the time of writing, to the best of our knowledge, there are no other experimental results available yet.

In this paper, we aim at improving the state-of-the-art in Thai semantic similarity. In an iterative process, we try different methods to this end, and combinations of those methods. The methods include: (i) instead of using pretrained models, train models ourselves on a Thai Wikipedia corpus, (ii) combine the idea of self-trained models and applying tokenization to the dataset terms, (iii) use subword-unit embeddings instead of conventional word embeddings, and (iv) integrate information from structured or hybrid sources (WordNet and ConceptNet) with the embeddings. The remainder of this section contains the evaluation results and their interpretation.

For clarity, we organize both the *evaluation setup* (Section Evaluation setup) as well as *evaluation results* (Section Evaluation results) according to the four approaches mentioned above.

### Evaluation setup

This section contains details on the *evaluation setup*, including the setup of the evaluation tool, and the configurations used in the experiments.

#### Self-trained models

The first step in embedding model training is the selection and preprocessing of an appropriate text corpus. We follow the conventional approach of other researchers, for example fastText [23], thai2vec, and Kyubyong vectors [17], and use Thai Wikipedia [51] as corpus. After downloading the XML-formatted dump, we extract the plain text with a Python script using the lxml library and regular expressions. Then we apply the state-of-the-art deepcut tool to segment the text into words which can be used as input for the word embedding algorithms. Deepcut [24] is a recent open source project which applies deep learning, and reaches 98.1% F1 on the BEST dataset for Thai word tokenization. The resulting plain text corpus is about 872MB in size and contains 56.4M tokens.

Then, we train word2vec and fastText (in this first experiment *without* subword-information) with the popular Gensim [52] library. The following settings are used for both word2vec and fastText: *word window size*: 5, *embedding vector size*: 300, *number of negative samples*: 5, *min. word frequency in corpus*: 2. The self-trained models are found online: word2vec models [53] and fastText models [54].

We experiment both with the skip-gram and continuous-bag-of-words (CBOW) algorithms.

The self-trained models are compared with the best-performing pretrained model from previous work [18] as baseline, which was thai2vec [15], model v0.1. Thai2vec was trained on Wikipedia with word2vec, and applied a dictionary-based word segmentation algorithm. As another baseline we add the pretrained fastText model to show the improvements of a self-trained model with a state-of-the-art tokenizer over the stock embedding. The pretrained fastText model is available online [14], where fasttext.cc provides models for 157 languages, trained with the CBOW algorithm, 300 dimensions, and a context window of 5 words.

#### Self-trained models and deepcut

Here, we use the same settings as in the first experiment, except for one aspect: aiming to reduce the number of out-of-vocabulary words, we apply the deepcut tokenizer also to the dataset terms within the evaluation tool. If a dataset term is not in the vocabulary of the model, when the evaluation tool splits it into its parts (if any) with deepcut. Finally, the term is represented by the sum of the vectors of the parts.

#### Subword-unit embeddings

A conceptual extension to splitting words with a tokenizer is the training of subword-unit embeddings, which in contrast to traditional embeddings, do not operate on a word, but on a character n-gram basis. We make use of two types of such embeddings which were introduced in Section Models and algorithms, namely BPEmb and fastText (with the subword feature).

BPEmb provides pretrained subword embeddings, with different options regarding vocabulary size (between 1000 and 200.000), and vector dimensionality (50, 100, 200, 300). In the evaluations we experiment with 300-dimensional vectors and different vocabulary sizes. In order to evaluate BPEmb, we use its .embed() function on all dataset terms to create an embedding vector for each term. After saving those vectors using the standard GloVe/.txt embedding format, we can feed them as input to the evaluation tool.

For fastText with subword feature we resort to a default setting, using the skip-gram algorithm, a word window of 5 words, and 300-dimensional vectors. Those vectors are self-trained on Thai Wikipedia with Gensim.

Finally, we experimented with stacking BPEmb and fastText vectors, so that in the example of stacking a 300-dim. BPEmb vector and a 300-dim. fastText word vector leads to a 600-dim. word representation.

#### Integration with WordNet and ConceptNet

As a last step we integrate the best-performing embeddings (ie. subword unit embeddings) with structured/ontological data. Such integration helped the top contenders in the SemEval2017 Task 2 challenge on semantic similarity. As structured (and hybrid) data sources we use both Thai WordNet and ConceptNet. WordNet’s *path_similarity* function provides a similarity score for two terms, which we first test in isolation, and then integrate it with the word embedding score using the two methods discussed in Section Implementation. Also the ConceptNet Numberbatch word vectors we first apply in isolation, and then in an ensemble with fastText.

As discussed in Section Implementation, Eq 1 uses the *α* coefficient to determine the weight of the embeddings and of the structured source in the final result. We experiment with *α* values in the interval of [0, 1] with a step-size of 0.05.

### Evaluation results

This subsection presents the evaluation results and the discussion of results for the four strategies to improve the state-of-the-art in Thai semantic similarity.

#### Self-trained models

Table 2 compares the results of the self-trained models with the baseline results from previous work. The self-trained fastText model outperforms the baseline on all datasets except TWS-65, which is by far the smallest dataset. The word2vec models are mostly about on par with the baseline, and the skip-gram (SG) variant performs better than continuous-bag-of-words (CBOW). As expected, one of the main problems identified in previous work, ie. the high number of OOV terms, remains. The default strategy in the evaluation tool is to replace those terms with average vectors. OOV words occur for example because the tokenization algorithm splits corpus terms into constituents which do not align with the dataset words, esp. in a language like Thai where most basic terms are compound from smaller units and where it is not always clear and agreed how to perform tokenization.

**Table 2:** Evaluation metrics Spearman *ρ* (S), Pearson *ρ* (P) and Harmonic Mean (HM) of the two–for the self-trained models and the pretrained baselines. Further, the ratio of OOV words (%OOV).

Model	TH-WordSim-353				TH-SemEval-500				TH-SimLex-999				TWS-65			
	S	P	HM	%OOV	S	P	HM	%OOV	S	P	HM	%OOV	S	P	HM	%OOV
w2v-300-SG	0.371	0.317	0.342	25.8	0.312	0.278	0.294	30.8	0.399	0.431	0.415	20.0	0.434	0.358	0.392	16.9
w2v-300-CBOW	0.310	0.285	0.297	25.8	0.263	0.252	0.257	30.8	0.304	0.357	0.328	20.0	0.419	0.404	0.411	16.9
fastText-300-SG	0.448	0.376	**0.409**	25.8	0.416	0.353	**0.382**	30.8	0.419	0.454	**0.436**	20.0	0.505	0.422	0.460	16.9
Baseline: fasttext	0.182	0.179	0.181	42.1	0.175	0.202	0.187	53.2	0.201	0.251	0.223	35.6	0.203	0.147	0.170	44.6
Baseline: thai2vec	0.384	0.331	0.356	18.4	0.317	0.261	0.286	34.1	0.359	0.443	0.397	7.8	0.505	0.504	**0.505**	7.7

In summary, the findings here are that a self-trained models, especially fastText, outperform the baseline, but not by a large margin. This results from the high fraction of OOV-terms in the basic version of the self-trained models. As a remark, in the table we give the ratio of OOV words in the dataset, the fraction of word pairs which have at least one OOV word in them is higher (up to two times).

#### Self-trained models and deepcut

In this set of experiments, we aim to reduce the number of OOV words in the models by applying the deepcut tokenization algorithm not only to the corpus, but also to the dataset terms. Table 3 provides an overview of the results, and shows large improvements with regards to the evaluation metrics. The self-trained fastText model now reaches around 0.6 for the harmonic mean of Pearson and Spearman *ρ* for all datasets. The rate of OOV words could be reduced drastically to between 0.0% (TWS-65) and 4.5% (TH-SemEval-500).

**Table 3:** Evaluation metrics Spearman *ρ* (S), Pearson *ρ* (P) and Harmonic Mean (HM) of the two–for the self-trained models and the pretrained baselines, *with deepcut applied to the datasets terms*. Further, the ratio of OOV words (%OOV).

Model	TH-WordSim-353				TH-SemEval-500				TH-SimLex-999				TWS-65			
	S	P	HM	%OOV	S	P	HM	%OOV	S	P	HM	%OOV	S	P	HM	%OOV
w2v-300-SG	0.547	0.531	0.538	1.1	0.538	0.557	0.547	4.5	0.540	0.619	0.577	0.6	0.568	0.590	0.579	0.0
w2v-300-CBOW	0.442	0.439	0.440	1.1	0.446	0.459	0.453	4.5	0.442	0.509	0.473	0.6	0.477	0.490	0.483	0.0
fastText-300-SG	0.625	0.589	**0.607**	1.1	0.631	0.638	**0.635**	4.5	0.553	0.626	**0.587**	0.6	0.676	0.647	**0.661**	0.0
Baseline: fastText	0.347	0.363	0.355	9.2	0.371	0.368	0.369	22.0	0.410	0.486	0.445	10.3	0.252	0.200	0.223	16.9
Baseline: thai2vec	0.471	0.433	0.451	3.3	0.425	0.363	0.392	16.0	0.432	0.518	0.471	1.3	0.530	0.589	0.558	0.0

Applying the deepcut tokenization to the datasets also helped to improve the scores for the two baselines, but for those a significant amount, up to 22.0% (for *Baseline: fastText (pretr.)*), of OOV words remain—because those pretrained models applied other tokenization algorithms in corpus preprocessing. When using this approach in semantic similarity, or any other application with corpus and target text, our results show that it is important to use the same tokenization algorithm for both (in our case text corpus and dataset terms).

As a remark, splitting words into parts is clearly not always optimal—as often the meaning of words is distinct from just the combination of the meanings of the word parts—but obviously this approach is better than using on average vector over the dictionary (which is the default strategy in the evaluation tool for OOV words).

In general, the improving results are in line with Table 2, fastText-SG outperforms word2vec-SG, which in turn yields better results than word2vec-CBOW. The vector dimension hyperparameter has little impact.

#### Subword-unit embeddings

Table 4 shows that using the fastText subword feature brings consistent improvements over the deepcut approach. The average score over all datasets is now around 0.66, the problem of OOV-words is solved, and also the additional step of applying deepcut to the dataset terms is not necessary any more.

**Table 4:** Overview of results for BPEmb (various settings), fastText embeddings, and stacked embeddings; with comparison to the baselines.

Model	TH-WordSim-353			TH-SemEval-500			TH-SimLex-999			TWS-65		
	S	P	HM	S	P	HM	S	P	HM	S	P	HM
BPEmb-1K-300	0.237	0.272	0.253	0.312	0.355	0.332	0.309	0.436	0.361	0.124	0.126	0.125
BPEmb-5K-300	0.450	0.479	0.464	0.495	0.544	0.518	0.446	0.573	0.502	0.664	0.599	0.630
BPEmb-10K-300	0.551	0.547	0.549	0.557	0.596	0.576	0.479	0.581	0.525	0.713	0.664	**0.688**
BPEmb-25K-300	0.558	0.551	0.555	0.582	0.602	0.592	0.517	0.599	0.555	0.696	0.673	0.684
BPEmb-50K-300	0.580	0.544	**0.561**	0.588	0.603	**0.595**	0.521	0.614	**0.564**	0.684	0.672	0.678
fastText (w. subword units)	0.634	0.620	**0.627**	0.693	0.684	**0.689**	0.583	0.650	0.615	0.703	0.711	0.707
stacked (fastText and BPEmb-50K-300)	0.620	0.599	0.609	0.637	0.631	0.634	0.606	0.667	**0.635**	0.712	0.746	**0.729**
Baseline: fasttext (pretrained)	0.182	0.179	0.181	0.175	0.202	0.187	0.201	0.251	0.223	0.203	0.147	0.170
Baseline: thai2vec	0.384	0.331	0.356	0.317	0.261	0.286	0.359	0.443	0.397	0.505	0.504	0.505

In our experiments the results for BPEmb are generally a bit lower than for fastText. However, BPEmb has the advantage of using a very small embedding model (depending on selected vocabulary size), which may be especially useful in situations of limited resources (for example in a mobile phone application). While for a BPEmb vocabulary size of 1K the results are poor, a model with only the 5K most frequent subword parts and words already provides a decent representation.

Stacking BPEmb and fastText embeddings led to mixed results, depending on the dataset. For two datasets the scores improve over fastText alone, so depending on the application, stacking is definitely an interesting option to experiment with.

#### Integration with WordNet and ConceptNet

Table 5 contains two main parts, the first part presents the Thai WordNet-related results, and the last three rows are results for ConceptNet Numberbatch. Within these parts, first we provide evaluation results for the structured/hybrid method in isolation, and then in an ensemble with BPEmb and fastText (with subword information).

**Table 5:** Overview of results for combining subword embeddings with structured and hybrid sources (WordNet and ConceptNet Numberbatch). M1 refers to *Method 1* from Section Implementation, M2 to *Method 2*.

Model	TH-WordSim-353			TH-SemEval-500			TH-SimLex-999			TWS-65		
	S	P	HM	S	P	HM	S	P	HM	S	P	HM
WordNet-only M1 + Simple variant	0.216	0.318	0.257	0.280	0.328	0.302	0.282	0.574	0.378	0.405	0.450	0.427
WordNet-only M1 + Most-Similar	0.265	0.359	0.305	0.355	0.393	0.373	0.350	0.596	0.441	0.567	0.583	0.575
WN-only without OOV—Simple variant	0.240	0.370	0.291	0.435	0.500	0.466	0.326	0.648	0.434	0.430	0.490	0.458
WN-only without OOV—Most-similar	0.384	0.443	0.412	0.567	0.609	0.587	0.439	0.708	0.542	0.604	0.634	0.619
M1 + WN Most-Similar + BPEmb-25K-300	0.571	0.556	**0.564**	0.616	0.630	**0.623**	0.569	0.677	**0.619**	0.808	0.755	**0.780**
M2 + WN Most-Similar + BPEmb.25K-300	0.568	0.553	0.561	0.601	0.623	0.612	0.560	0.664	0.608	0.828	0.758	0.791
Table 4: BPEmb.25K.300	0.558	0.551	0.555	0.582	0.602	0.592	0.517	0.599	0.555	0.696	0.673	0.684
M1 + WN Most-similar + fastText (subw.)	0.645	0.622	0.634	0.713	0.700	0.706	0.610	0.696	**0.650**	0.799	0.774	**0.787**
M2 + WN Most-similar + fastText (subw.)	0.653	0.620	**0.636**	0.721	0.704	**0.712**	0.608	0.690	0.646	0.800	0.774	**0.787**
Table 4: fastText (subw.)	0.634	0.620	0.627	0.693	0.684	0.689	0.583	0.650	0.615	0.703	0.711	0.707
ConceptNet Numberbatch-Only	0.617	0.595	0.606	0.664	0.687	0.675	0.639	0.696	0.666	0.898	0.896	0.897
M1 + ConceptNet + fastText (subw.)	0.709	0.668	**0.688**	0.780	0.759	**0.769**	0.692	0.743	**0.717**	0.899	0.901	0.900
M2 + ConceptNet + fastText (subw.)	0.707	0.667	0.687	0.777	0.755	0.766	0.692	0.743	**0.717**	0.900	0.901	**0.901**

The results for using WordNet *path_similarity* alone as measure of semantic similarity are in the range of pretrained embeddings (baseline), this is 0.25−0.57. We can see that the “most similar” variant clearly performs better than the “simple variant”. Therefore for combining WordNet with embeddings we focus on the “most similar” variant. See Section Implementation for details on those variants. Some dataset terms were not found in Thai WordNet, the number of OOV word pairs per dataset is as follows: 97 of 353 for TH-WordSim-353, 274 of 500 for TH-SemEval-500, 222 of 999 for TH-SimLex-999, and 10 out of 65 for TWS-65. Remember that for OOV word pairs the evaluation tool defaults to the mean *path_similarity* value over the dataset. In line with Netisopakul et al. [18]—out of interest—we present the results for WordNet considering only word pairs which have WordNet similarity scores. For those, the metric shows values between 0.41 and 0.62. This shows that scores for WordNet in isolation are below the scores of the best results for embeddings in isolation, even when considering only in-vocabulary terms. In the following we investigate if integrating the two approaches yields benefits.

In the second partition of the table we combine BPEmb and WordNet and compare the results to BPEmb alone (taken from Table 4). We can see that the combination provides clear benefits on all datasets. The setting *M1 + Most-Similar + BPEmb-25K-300* yields the best results and therefore the strongest improvements. For example for TH-SemEval-500 this setting provides a score of 0.62 (vs. 0.59 of BPEmb-25K-300), and for TH-SimLex 0.62 versus 0.55. The largest gain is achieved for the small TWS-65 dataset, with 0.78 versus 0.68.

Then we integrate WordNet and fastText (with subword information) and again experience benefits. The setting *M2 + Most-Similar + fastText (subw.)* provides the highest scores. Those results are also the best results achieved when ensembling WordNet with word embeddings. The improvements over the word embedding-only baseline are in a similar range as in partition two about BPEmb and WordNet.

In part two of the table (the last three rows) we present the evaluation metrics for ConceptNet Numberbatch. We see that ConceptNet by itself (*ConceptNet Numberbatch-Only*, using the ConceptNet OOV strategy) already delivers results which clearly outperform fastText (subw.) on two datasets. It should be noted that the ontological information included in ConceptNet seems to help with the difficult TH-SimLex-999 dataset and its strict definition of similarity. The best results overall are achieved by the ensemble of ConceptNet Numberbatch and fastText (subw.), with a large improvement versus WordNet and fastText, and results such as 0.77 for TH-SemEval-500 or 0.90 for TWS-65.

With regards to the *α* coefficient from Eq 1, we found that the optimal setting for WordNet experiments is in the range of 0.6−0.8, depending on the dataset. We recommend a value of 0.7, which means that the word embeddings contribute 70% to the final score, while WordNet contributes 30%. For ConceptNet on the other hand, we suggest *α* to be between 0.1−0.5, depending on the dataset, so that ConceptNet usually has a larger impact than traditional word embeddings. The experiments show that slight changes of *α* around the optimal value have only little impact on the metric. This means, even if the optimal *α* should be 0.6 for a dataset, 0.7 still gives close to optimal performance.

Finally, we conducted an error analysis aiming to analyze cases where the best performing method (ConceptNet Numberbatch and fastText) still fails. To this end, for any of the four datasets, we ordered the term pairs by *difference* in Spearman rank between the manual (gold standard) dataset and the model similarities—and then investigated term pairs where that difference was largest, i.e. that were misclassified by the model. One category of words where the model struggled are some (near-)synonyms, which were not detected as very similar. Our intuition is that in these cases assembling the words from their subword components lead to suboptimal vector representations. Furthermore, the model sometimes determines word pairs to be more similar than humans do, esp. for antonyms and contextually related words. As word embeddings models typically learn their embeddings from the contextual use of words, this behaviour can be expected. Esp. for datasets like SimLex-999, which clearly distinguish similarity from relatedness, this source of errors could be observed.

#### Summary and discussion

**Fig 3:**
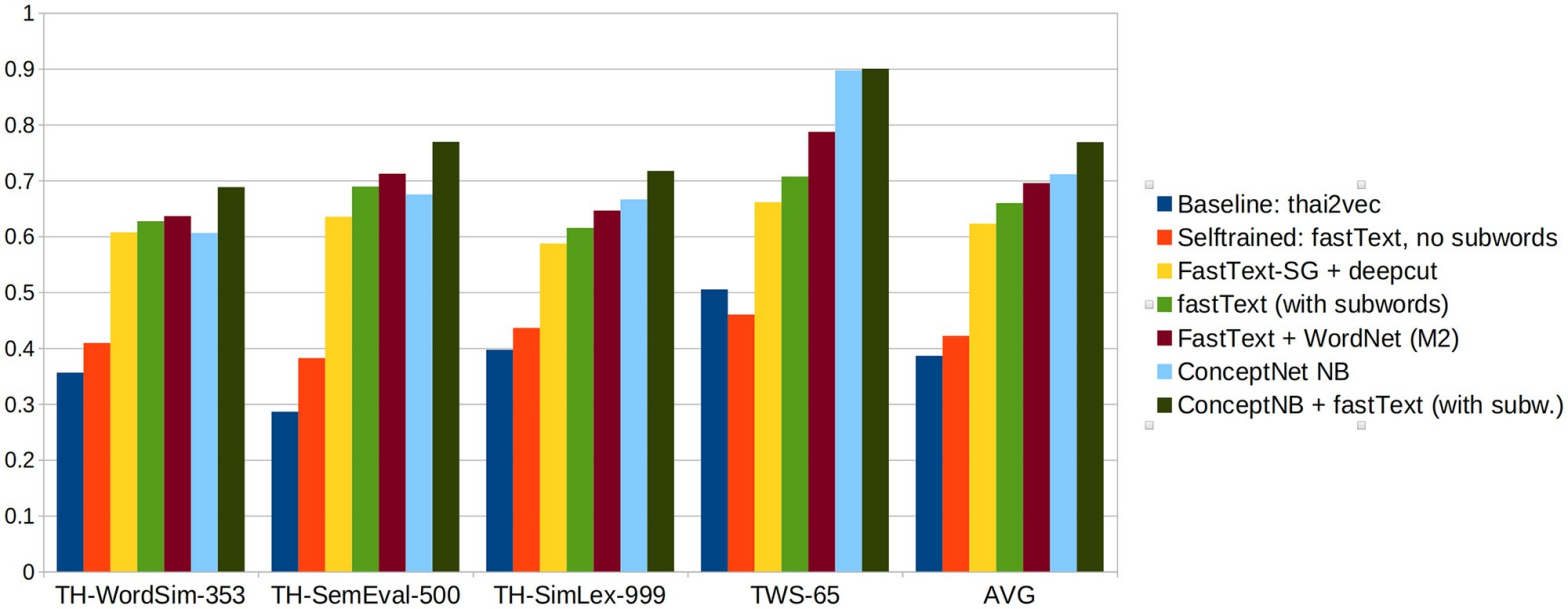
Overview of results. Comparing the baseline from previous work (*Baseline: thai2vec*) with the various approaches implemented in this work.

Fig 3 summarizes the main evaluation results. It compares the initial baseline (*Baseline: thai2vec*) to representatives of the approaches tested and evaluated in this work. Those approaches include: (i) training our own model on Thai Wikipedia (represented in the figure by model *Self-trained: fastText, no subwords*), (ii) self-trained models plus the application of the deepcut tokenizer on the dataset terms (*fastText-SG + deepcut*), (iii) using subword-unit embeddings instead of traditional word embeddings (*fastText with subwords*), (iv) combining the subword-unit embeddings with a structured data source (*fastText + WordNet (M2)*), (v) and finally ConceptNet Numberbatch by itself (*ConceptNet NB*), and in an ensemble with fastText (*ConceptNet NB + fastText with subwords*).

Fig 3 shows the results for each dataset, and the average over the four datasets. We can see clearly how the approaches described allow to surpass the baseline starting point by a large margin. Esp. the introduction of subword embeddings proves to be very helpful, as it is an effective way to mitigate the OOV problem. In Section Introduction we discussed cases where Thai words are compound from basic words (for example “student” is “person+learn”), which intuitively explains why the Thai language may be well suited for subword embeddings that learn embeddings for the constituents of words.

To summarize the approaches we applied to solve the high fraction of OOV terms in Thai language, there are three strategies: Firstly, to apply the same tokenization method (eg. Deepcut) on both the corpus and the dataset strings. This eliminates a large portion of OOV words as well as improves the results of the evaluation metrics, as can be seen in Tables 2 and 3. The second strategy is to use subword embeddings such as fastText (with subword units) and BPEmb to entirely mitigate the OOV problem. Thirdly, the combination of both distributional semantic information and ontological information (such as WordNet or ConceptNet) into an ensemble proved to yield the best results.

With regards to approach (iv), although WordNet by itself does not provide very high *ρ* scores (see Section Integration with WordNet and ConceptNet), when combined with embeddings it helps improve the results further. But the best results overall are achieved using ConceptNet in combination with fastText (with subword information).

Although the dataset translations are not directly comparable, let us contrast our results with the state-of-the-art in other languages. SemEval-2017 (task 2) organized a competition on semantic similarity for the SemEval-500 dataset in 5 languages. As stated, SemEval-500 is the most recent, and a rather difficult, dataset. While most of the 24 competing systems in SemEval-2017 did not reach the 0.70 mark in any of the five languages, the competition winners reached 0.79 for English, 0.71 for Farsi, 0.70 for German, and 0.74 for Spanish and French. Given that Thai is a very difficult language to handle for NLP, with no word boundaries and complex word formation, we think that our result of 0.77 for SemEval-500 is remarkable and the approach is competitive also beyond the boundaries of Thai language. Also in comparison with the state-of-the-art of the difficult SimLex-999 English dataset [55], our method is very promising.

Finally, regarding pros and cons of the discussed methods, fastText (with subword units) gives the best individual results for traditional word embeddings, BPE embeddings provide both good results and a low memory footprint, and WordNet can help to raise the scores when combined with embedding models, but by itself lacks coverage of vocabulary. The hybrid ConceptNet embeddings, which contain both ontological and distributional information, esp. in combination with fastText, allow to reach the best results.

As discussed in Section Datasets, the SimLex-999 dataset captures *similarity*, as opposed to *relatedness*, of terms. Kiela et al. [56] stated that corpus-driven methods generally learn both similarity and relatedness reasonably well, but in their experiments they found better results for relatedness datasets. This corresponds to our results, where TH-SimLex-999 gave the lowest score when using the fastText (with subword units) embeddings. ConceptNet Numberbatch on the other hand provides much better results on TH-SimLex-999 than fastText (0.67 vs 0.61). This indicates, that the integration of ontological knowledge into ConceptNet Numberbatch is particularly helpful to capture a more formal and strict definition of similarity.

## Conclusion

In this paper we analyze various strategies to raise the state-of-the-art in Thai semantic similarity as measured on four existing datasets for Thai language: TH-WordSim-353, TH-SemEval-500, TH-SimLex-999, and TWS-65. Word embedding models are frequently used on the semantic similarity task, and vice versa, the datasets provide a way to intrinsically evaluate the quality of the embedding models. In the process, we solve the issue of out-of-vocabulary dataset words reported in Netisopakul et al. [18], firstly by training our own models and applying state-of-the-art word tokenization on both the corpus and the dataset terms. Even more effective and easier to implement is the application of subword-unit embeddings. Finally, inspired by related work, we combine embedding models with information from structured and hybrid sources (WordNet and ConceptNet) to further improve the results. Overall, we achieve an average harmonic mean of Pearson and Spearman correlation (our evaluation metric) over the four datasets of 0.77, as compared to 0.38 in previous work.

Our work is the first comprehensive study of semantic similarity for Thai language and the problems specific to Thai. The contributions of this work are as follows: (i) The main contribution is improving the state-of-the-art in Thai language semantic similarity by a large margin. For any of the four existing word pair datasets we achieve a large improvement over the previous baseline. (ii) Analysis of the capabilities and pros and cons of different strategies and embedding models on Thai semantic similarity. (iii) Presenting a method to integrate word embeddings with structured sources (like WordNet or ConceptNet) for the semantic similarity task in situations with OOV words occurring in structured sources. (iv) The provision of an evaluation tool to easily test new embedding models with the Thai datasets.

In future work, there are different angles to potentially improve the results further. Firstly, the models can be trained on larger text corpora or a combination of corpora. Secondly, other structured sources, for example BabelNet can be evaluated. Finally, one can find ways to combine more than two sources into one model, for example both WordNet and BabelNet, and multiple embedding types in an ensemble learning approach. Another interesting aspect of future work is to experiment with extrinsic evaluation measures, ie. to evaluate Thai word embeddings on various NLP downstream tasks.
